# Supplementary material for: Interaction of MRPL9 and GGCT Promotes Cell Proliferation and Migration by Activating the MAPK/ERK Pathway in Papillary Thyroid Cancer
Source: Int J Mol Sci. 2022 Oct 9;23(19):11989. doi: 10.3390/ijms231911989 (PMC9570013; doi:10.3390/ijms231911989)
Supplement: Supplementary file 1 [file ijms-23-11989-s001.zip › ijms-1903854-supplementary.pdf]

Interaction of MRPL9 and GGCT Promotes Cell Proliferation and Migration by Activating the MAPK/ERK Pathway in Papillary Thyroid Cancer

**Hui-Min Zhang [1,†], Zi-Yi Li [1,†], Zhou-Tong Dai [1,2], Jun Wang [1], Le-Wei Li [1], Qi-Bei Zong [1], Jia-Peng Li [1],
Tong-Cun Zhang [1,*] and Xing-Hua Liao [1,*]**

1 Institute of Biology and Medicine, College of Life and Health Sciences, Wuhan University of Science and Technology, Wuhan 430070, China; huiminzhang@wust.edu.cn (H.-M.Z.); lzy954599@126.com (Z.-Y.L.); zhoutongdai@126.com (Z.-T.D.); 201818703005@wust.edu.cn (J.W.); leweili@wust.edu.cn (L.-W.L.); qibeizong@wust.edu.cn (Q.-B.Z.); jiapengli@wust.edu.cn (J.-P.L.)
2 Department of Obstetrics and Gynecology, Tongji Hospital, Tongji Medical College, Huazhong University of Science and Technology, Wuhan 430030, China
* Correspondence: zhangtongcun@wust.edu.cn (T.-C.Z.); xinghualiao@wust.edu.cn (X.-H.L.)
† These authors contributed equally to this work.

Supplementary Material

Table S1. Base sequence information.

Name	Sequence of bases	product size (bp)
qPCR-GGCT-forward primer	TGGCAATTCCCAAGGCAAAAC	140
qPCR-GGCT-reverse primer	CCCCTTCTTGCTCATCCAGAG	
qPCR-MRPL9-forward primer	GTTACCAGAAGAGCCTATCACAC	77
qPCR-MRPL9-reverse primer	CTCTCACAGTATCAAGCCCATTT	
qPCR-Actin-forward primer	CATGTACGTTGCTATCCAGGC	250
qPCR-Actin-reverse primer	CTCCTTAATGTCACGCACGAT	
PCR-GGCT-forward primer	ATGGCCAACTCGGGCTGC	567
PCR-GGCT-reverse primer	CTAAAGAGTTTGTGTTTCCCCCTTT	
PCR-MRPL9-forward primer	ATGGCGGCGCCCGTTGTCA	804
PCR-MRPL9-reverse primer	TTAGATCTGGGGGCTGGTGGGGG	
shGGCT	GCTGGAGTATCAAGAGAAGTT	
shMRPL9	GTGACCTGGTCTCAGTGAA	

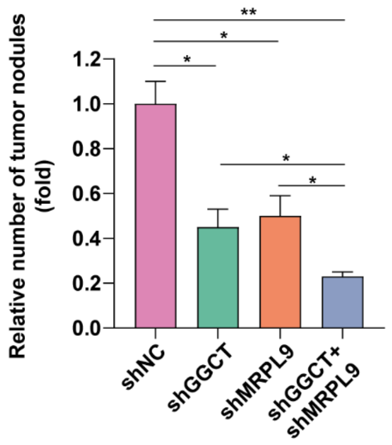

Figure S1. HE staining was performed on the lung tissue of nude mice, and the lung tumor nodules were counted (n=3). One-way analysis of variance (ANOVA) was used with Tukey's post hoc test. “*” represents $P \leq 0.05$, “**” represents $P \leq 0.01$.
